# Selected proceedings of the 2009 Summit on Translational Bioinformatics

**DOI:** 10.1186/1471-2105-10-S9-I1

**Published:** 2009-09-17

**Authors:** Yves A Lussier, Indra Neil Sarkar

**Affiliations:** 1 Center for Biomedical Informatics and Section of Genetic Medicine of the Department of Medicine, Institute for Translational Medicine, Institute for Genomics and Systems Biology, Computational Institute, and Ludwig Center for Metastasis Research; Cancer Research Center, University of Chicago, 5841 South Maryland Ave, Room N-660B, Mail Code 6091, Chicago, IL 60637, USA; 2 Center for Clinical and Translational Science, University of Vermont, Burlington, VT 05401, USA

## Background

"It is the responsibility of those of us involved in today's biomedical research enterprise to translate the remarkable scientific innovations we are witnessing into health gains for the nation [1]." The First Summit on Translational Bioinformatics (held in 2008) was organized in response to the call for translational bioinformaticians that was issued by the Director of the NIH, at a time there was no national annual conference or symposium for the presentation and discussion of research work in Translational Bioinformatics.

This Second Summit built on the success of the first Summit on Translational Bioinformatics, highlighting the multidisciplinary nature of this rapidly maturing research field and providing the opportunity to forge new transdisciplinary collaborations as the finest minds of the academia, industry, government and non-profit sector are brought together. Reflecting on the unique position of translational bioinformatics in medical research and healthcare management, the 2009 Scientific program Committee designed a comprehensive program comprised of the four original tracks (#1–4) and with two new ones (#5,6):

1 – Informatics methods for the analysis of molecular and clinical measurements,

2 – Computational approaches to finding mechanisms and therapies for diseases,

3 – Relating and representing phenotypes and diseases,

4 – Dissecting disease through the study of organisms, evolution and taxonomy,

5 – Informatics concepts, tools, and techniques to enable integrative translational research,

6 – Informatics methods in genetics discoveries and clinical practice.

## Workshop program

Three hundred and fifty attendees, from 201 distinct institutions or companies covering 36 states in the US and 17 other countries, met over three days in San Francisco, CA. Sixty-three papers and thirty-seven abstracts were submitted for oral presentation to the Summit, of which 39 and 28 were respectively accepted for oral presentation by the Scientific Program Committee (Additional file 1). In addition to these papers, 3 tutorials, 64 posters and 8 panels were presented.

The opening session keynote was by Dr. Lynn H. Vogel, Vice President and Chief Information Officer at The University of Texas M.D. Anderson Cancer Center in Houston. Dr. Vogel presented the concepts and investments underway at the M.D. Anderson Cancer Center for "Building an IT Architecture to Facilitate Personalized Medicine". The second keynote was Dr. Andrea Califano, Professor of Biomedical Informatics at Columbia University, where he is currently Director of the Center for the Multiscale Analysis of Genetic Networks (MAGNet – one of seven NIH-funded National Centers for Biomedical Computing), Associate Director for Bioinformatics of the Herbert Irving Comprehensive Cancer Center (HICCC), and co-Director of the Center for Computational Biology and Bioinformatics (C2B2). He presented on "Using Cancer Interactome for therapeutic target and associated biomarker discovery". The closing session keynote presentation was delivered by Dr. Russ B. Altman, Professor and Chair of Bioengineering, Professor of Medicine, Genetics, and Computer Science (by courtesy), Stanford University, and Principal Investigator of Simbios, one of the National Centers for Biomedical Computing. His presentation highlighted a year-in-review of the literature in Translational Bioinformatics.

Slides from most tutorials, papers, and panels are publicly available at 

## Summary of the selected contributions

The seventeen papers selected for this supplement to *BMC Bioinformatics *are extended and improved versions of the best papers accepted to the 2009 Summit of Translational Bioinformatics. In the following paragraphs, we briefly describe them.

Six of these papers deal with the science of network analyses applied to the Translational Bioinformatics field. Chang and Ramoni show how Bayesian Networks (BN) can improve on current microarray analyses by generating a predictor that does not require the biased adjustment of parameters of the current state if the art. An unbiased BN predictor of aortic aneurysm diagnosis is provided as proof of concept [2]. Lee *et al*. show how protein interactions and gene expression can be combined for predicting novel joint networks ignored by array analyses alone[3]. Bhavnani *et al. *provide a molecular classification of renal diseases showing gene expression similarities rather than morphological similarities, perhaps illustrative of shared expression underpinning organ-specific phenotypic networks [4]. Butte's group illustrates how non-trivial correlations between two physiological measurements in intensive care unit can be more reliable for prognosis than single measurements or laboratory values suggesting an unbiased and systematic approach to discovery of novel complex prognosis markers [5]. Dean *et al. *demonstrated how evolutionary conserved binding sites of transcriptional factors used jointly with expression profiling can be used to subsequently construct cis-regulatory expression modules in osteocytes [6]. Yang and Lussier propose a novel and robust algorithm to identify epigenetic regulation derived from co-expression networks and epigenetic targets in the literature, with a specific evaluation in terms of significance and accuracy in the context of Acute Lymphobalstic Leukemia [7].

Two papers are in the area of decision making relying on genomic or complex multiscale analyses. The PAPAyA framework integrates the complexity of multiscale analyses inclusive of clinical prognosis, histopathological morphology and genomic measurements to facilitate multiscale biomarker development, hypothesis generation and decision support [8]. Overby provides a framework for question answering with genomic and clinical knowledge from the Unified Medical Language System [9].

Three papers were highlighted in the area of large-scale repositories in support of genomic medicine. Ohno-Machado's group create a comprehensive and consistent annotation system for the context in which samples are annotated in the NIH Gene Expression Omnibus database (GEO), inclusive of tissue, cell line, histopathological type, and subject characteristics such as demographics, treatment, or survival. They report an implementation of over 6,000 samples extracted from GEO [10] and inter-rater agreement of 92% [11]. Tarczy-Hornoch's group created SNPit, a database that provides some guidance on the variability of SNPs as an indicator of an adequate measure for clinical relevance [12].

Three papers focused on phenotypic representation, their ontologies and their coding in narratives. South *et al. *provided a practical approach to text processing of phenotypic information in clinical records [13], while Wang, Hripcsak and Friedman provide an algorithm based on mutual information to disclose interdependencies that may not be linear between environmental (medication) and phenotypic data [14]. Musen's group provides a novel algorithm for term-based ontology annotation that performs better than the NIH gold standard MetaMap[15].

Finally, three papers were chosen in the unrelated areas of fMRI, structural biology and rule learning. Jin *et al. *demonstrated a feature-learning algorithm to identify patterns of voxels in fMRI associate to phenotypic classification [16]. Lustgard used an ontology-anchored set of known biomarkers in order to improve machine learning algorithms designed to discover novel ones in a proteomic datasets [17]. Shivakumar and Krauthammer use structural similarity to identify substitute sensitivity profiles for medications that are not included in cancer screens, aiming to potentially increase the relevance of existing anti-cancer drug screens [18].

The next Summit on Translational Bioinformatics will be held in 2010 in San Francisco.

## Competing interests

The authors declare that they have no competing interests.

## Authors' contributions

Both authors wrote and approved the manuscript. Lussier was the Chair of Scientific Program Committee for the 2009 AMIA Summit on Translational Bioinformatics; Sarkar was the Vice-Chair of the Scientific Program Committee and the Chair of the Poster Program Committee for the 2009 AMIA Summit on Translational Bioinformatics.
